# Emerging Trends in Thermo-Optic and Electro-Optic Materials for Tunable Photonic Devices

**DOI:** 10.3390/ma18122782

**Published:** 2025-06-13

**Authors:** Muhammad A. Butt

**Affiliations:** Institute of Microelectronics and Optoelectronics, Warsaw University of Technology, Koszykowa 75, 00-662 Warsaw, Poland; ali.butt@pw.edu.pl

**Keywords:** reconfigurable photonic devices, thermo-optic, electro-optic, Pockels effect, Kerr effect

## Abstract

Tunable photonic devices are increasingly pivotal in modern optical systems, enabling the dynamic control over light propagation, modulation, and filtering. This review systematically explores two prominent classes of materials, thermo-optic and electro-optic, for their roles in such tunable devices. Thermo-optic materials utilize refractive index changes induced by temperature variations, offering simple implementation and broad material compatibility, although often at the cost of slower response times. In contrast, electro-optic materials, particularly those exhibiting the Pockels and Kerr effects, enable rapid and precise refractive index modulation under electric fields, making them suitable for high-speed applications. The paper discusses the underlying physical mechanisms, material properties, and typical figures of merit for each category, alongside recent advancements in organic, polymeric, and inorganic systems. Furthermore, integrated photonic platforms and emerging hybrid material systems are highlighted for their potential to enhance performance and scalability. By evaluating the tradeoffs in speed, power consumption, and integration complexity, this review identifies key trends and future directions for deploying thermo-optic and electro-optic materials in the next generation tunable photonic devices.

## 1. Introduction

Photonic devices, which manipulate and control the propagation of light, are central to a wide range of modern technologies including optical communications, sensing systems, imaging platforms, and emerging fields such as quantum information processing [1,2,3,4]. As these technologies evolve, there is a growing need for optical components that can dynamically adjust their behavior in real time [5,6,7,8]. Devices with fixed optical properties are often insufficient for systems that require flexibility and adaptability. This increasing demand has led to the development of tunable photonic devices, which can modify their optical characteristics, such as the refractive index or transmission spectrum, in response to external stimuli [9,10,11]. The ability to reconfigure photonic devices on demand is essential for enabling multifunctional operation, enhancing system performance, and supporting scalable architectures in advanced applications like optical networks, beam steering, and signal modulation [12,13].

Magneto-optic materials are key enablers of tunable photonic devices, offering dynamic control over light propagation through magnetically induced changes in optical properties such as polarization and the refractive index. These materials are particularly valuable for applications requiring non-contact, localized tuning, such as optical isolators, modulators, and reconfigurable circuits [14,15]. However, their practical use faces several challenges. The magneto-optic effect is generally weak, often necessitating strong magnetic fields or long interaction lengths, which limits miniaturization. Additionally, integration with standard photonic platforms is complex, as many magneto-optic materials are not compatible with CMOS processes. The need for external magnets and the potential for magnetic interference further complicate device design [16].

Among the various mechanisms that enable tunability in photonic systems, the thermo-optic (TO) [17,18] and electro-optic (EO) [13,19] effects are particularly significant due to their strong influence on optical behavior and their compatibility with integrated device platforms. The TO effect refers to the variation in a material’s refractive index as a function of temperature, typically described by the TO coefficient [20]. This effect can be harnessed through localized heating techniques, allowing controlled changes in optical properties over time. The EO effect, on the other hand, involves a change in the refractive index in response to an applied electric field. This phenomenon can occur through either the linear Pockels effect or the nonlinear Kerr effect, depending on the symmetry and electronic structure of the material [21,22,23]. EO modulation is often much faster than TO tuning and is widely used in high-speed applications such as optical switches and modulators [24,25].

The effectiveness of tunable photonic devices is closely tied to the properties of the materials used [26,27,28,29,30]. An ideal material for TO [31,32,33] or EO tuning [34,35,36,37] should exhibit a large change in the refractive index with minimal energy input, low optical absorption, thermal and chemical stability, and compatibility with the fabrication processes used in photonic integration. For instance, silicon (Si) is a leading platform in integrated photonics due to its well-established manufacturing infrastructure and significant TO response [38,39,40]. However, Si lacks a linear EO effect due to its centrosymmetric crystal structure, limiting its use in high-speed modulation applications. This challenge has motivated the integration of EO active materials such as lithium niobate (LN), barium titanate (BTO), and organic polymers with Si to combine the benefits of both platforms [41,42]. In recent years, researchers have also begun exploring two-dimensional materials and hybrid perovskites for their promising optical properties and potential for scalable integration [20,43,44,45]. Perovskites have rapidly gained attention in photonics due to their exceptional optoelectronic properties, such as their high absorption coefficients, tunable bandgaps, and strong nonlinear optical responses [46,47]. These materials, characterized by their distinctive crystal structure (ABX_3_), offer great versatility through compositional engineering, enabling tailored optical behaviors for applications including lasers, light-emitting diodes, and photodetectors. In addition, emerging perovskite-based photonic devices demonstrate promising EO and TO tunability, making them attractive candidates for next-generation modulators and switches [48,49,50]. Despite challenges related to material stability and uniform thin-film fabrication, ongoing advances continue to improve their performance and integration potential in photonic circuits [51].

This review presents an in-depth analysis of the materials employed in tunable photonic devices that leverage TO and EO effects. It begins by elucidating the physical principles underpinning these phenomena and continues with a thorough evaluation of various material platforms, including conventional semiconductors, ferroelectric oxides, polymers, and emerging materials such as graphene, chalcogenides, and transparent conductive oxides. Each material is discussed concerning its tunability, compatibility with photonic integration, and performance trade-offs in specific applications. The review further explores recent advancements in material engineering techniques, such as nanostructuring, doping, and heterostructure fabrication, that have improved optical tunability and broadened functional capabilities. The primary objective of this work is to deliver a comprehensive and critical overview of the current and emerging materials landscape for tunable photonics, identifying both the present capabilities and the key challenges that must be addressed to enhance device performance and integration for future photonic systems.

## 2. Fundamentals of TO and EO Effects

Tunable photonic devices depend critically on materials for which the optical properties can be dynamically altered [30]. Among the most effective mechanisms for such modulation are the TO and EO effects [13,18]. These phenomena allow for the real-time control of key optical parameters such as the refractive index and absorption, which in turn enables dynamic tuning of the wavelength, phase, intensity, and polarization in integrated photonic circuits [52,53].

TO and EO tunable devices differ significantly in their modulation speeds due to the distinct physical mechanisms that they employ [54]. TO devices operate by exploiting the TO effect, where the refractive index of a material changes in response to temperature variations and is represented as follows:

$$\Delta n=\frac{dn}{dT}\Delta T$$

where $\frac{dn}{dT}$ is the thermo-optic coefficient (K^−1^), and ΔT is the temperature change. This process is typically achieved using integrated micro-heaters that locally raise the temperature of optical waveguides or resonators. However, the modulation speed of these devices is inherently limited by the slow nature of thermal diffusion and the thermal mass that must be heated or cooled [55]. As a result, TO devices generally exhibit modulation times in the range of microseconds to milliseconds, making them suitable for applications where tuning speed is not critical but where a broad tuning range and design simplicity are advantageous. In contrast, EO devices utilize the EO effect, particularly the Pockels effect in materials like lithium niobate, where an applied electric field induces an almost instantaneous change in the refractive index. The Pockel effect is defined as follows:

$$\Delta \left(\frac{1}{n^{2}}\right)=r_{ij}E_{j}$$

where *r_ij_* are the Pockels coefficients, and *E_j_* is the component of the applied electric field. Because this process is governed by fast electronic responses rather than slower thermal effects, EO devices can achieve modulation speeds on the order of nanoseconds or even picoseconds [56]. This makes them ideal for high-speed optical modulation, switching, and communications. However, they may require higher operating voltages and typically offer a more limited tuning range compared to TO devices.

TO and EO effects originate from fundamental material responses to thermal and electric stimuli, respectively. The TO effect is typically driven by temperature-induced changes in a material’s electronic polarizability, lattice expansion, and band structure, especially in semiconductors where bandgap narrowing alters the refractive index via the Kramers–Kronig relations [57]. In phase-change materials like Sb_2_S_3_, phase transitions between amorphous and crystalline states cause significant refractive index shifts due to structural and electronic changes. For EO effects, particularly the Pockels effect, the refractive index changes result from electric-field-induced lattice distortions and polarization changes in non-centrosymmetric materials such as lithium niobate [58]. Additionally, the electro-absorption effects and ion migration in materials like CuCrP_2_S_6_ offer alternative EO modulation pathways. These mechanisms highlight how the material structure, symmetry, and bonding dictate the optical response to thermal and electrical control.

Compared to magneto-optic (MO) and all-optical modulation techniques, EO and TO modulation offer improved integration with standard photonic platforms and more straightforward control schemes. However, while EO and TO approaches provide greater scalability and are often easier to fabricate, MO effects enable non-reciprocal behavior, and all-optical control allows ultrafast, low-energy switching without the need for electrical interfaces, though both often suffer from material or integration constraints.

### 2.1. TO Effect in Photonic Tuning

The TO effect refers to the change in a material’s refractive index due to temperature variation [33]. This relationship is quantified by the thermo-optic coefficient (TOC), represented mathematically as dn/dT. The TO effect is especially attractive for tunable photonic devices due to its broadband nature and compatibility with a wide range of materials [59]. In practical implementations, localized temperature control using integrated micro-heaters is employed to modulate the refractive index of photonic waveguides. This enables functionalities such as phase tuning, wavelength adjustment, and the detuning of optical resonators. Si photonics benefits significantly from this mechanism, with Si exhibiting a TOC of approximately 1.86 × 10^−4^ K^−1^ [60]. Although the response time of TO tuning is relatively slow, typically ranging from microseconds to milliseconds, it is widely used due to its design simplicity and low optical insertion loss. Beyond Si, materials such as polymers [33,61] and chalcogenide glasses [62,63] exhibit higher TOCs, making them suitable for more compact and power-efficient devices. Two-dimensional materials, including graphene oxide, are also gaining attention for their strong TO response and integration flexibility in photonic platforms [32,64].

**Figure 1:**
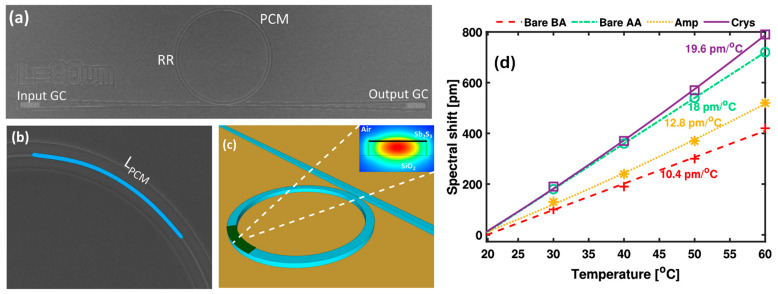
(**a**) SEM image of an microring resonator (MRR) with a PCM layer on top, (**b**) enlarged view of the PCM region, highlighted in blue, (**c**) schematic of a SiNx microring partially coated with Sb_2_S_3_, with an inset showing the cross-section and TE mode profile, and (**d**) transmission spectra of the MRR measured at various temperatures for four configurations: (**a**) SiN RR without PCM, before annealing, (**b**) Same RR after annealing, (**c**) RR with Sb_2_S_3_ in the amorphous state, and (**d**) RR with Sb_2_S_3_ in the crystalline state, with all spectra centered near 1550 nm [66].

Antimony sulfide (Sb_2_S_3_), a phase change material (PCM) with a wide bandgap, has been identified as a promising candidate for NIR tunable photonics due to its broadband optical transparency extending from the visible to the NIR spectrum [65]. Its suitability for integrated photonic applications has been explored through experimental analysis and device integration. Ilie et al. incorporated Sb_2_S_3_ onto a silicon nitride (SiN) photonic platform using a microring resonator (MRR) to investigate its thermally induced optical modulation capabilities [66]. Figure 1a,b display SEM images of a variation in the MRR, along with a close-up view of the PCM deposited on the arc section. Figure 1c presents a schematic of the SiNx RR, including a cross-sectional view that illustrates the fundamental TE optical mode. The thermo-optical behavior of Sb_2_S_3_ in the C-band was explored, focusing on its impact on high-density photonic applications. The temperature dependence of the RR resonance was studied between 20 °C and 60 °C using a Peltier stage, with measurements taken after 30 min of stabilization at each temperature. The temperature sensitivity of the bare SiNx RR’s resonance wavelength increased from 10.8 pm/°C before annealing to 18.0 pm/°C after annealing (red and green lines in Figure 1d). For the amorphous Sb_2_S_3_ state, the shift was 12.8 pm/°C, while the crystalline state exhibited a shift of 19.6 pm/°C. Despite changes in the thermo-optical properties of the ZnS-SiO_2_ cladding after annealing, the optical losses remain comparable to those of air-cladded devices [66].

Phase transitions between the amorphous and crystalline states of the material were utilized to achieve dynamic tuning of the resonator response, with extinction ratios reaching up to 18 dB in the C-band. TOCs were extracted for both states, measured at approximately 3.4 × 10^−4^ K^−1^ for the amorphous phase and 0.1 × 10^−4^ K^−1^ for the crystalline phase, reflecting a significant refractive index contrast. Additionally, permanent spectral trimming of the device was demonstrated through bidirectional tuning enabled by continuous-wave (CW) laser exposure in the range of −5.9 to 5.1 dBm. This process resulted in effective refractive index (n_eff_) modulation from +5.23 × 10^−5^ to −1.20 × 10^−4^. These findings confirmed the feasibility of using Sb_2_S_3_ for both reconfigurable and non-volatile photonic functionality. Potential applications include optically programmable memory, tunable synaptic elements for neuromorphic systems, and dense optical switching architectures within multilayer PECVD-based photonic integrated circuits (PICs) [66].

### 2.2. EO Effect in Photonic Tuning

The EO effect involves the modification of a material’s refractive index in response to an externally applied electric field [36]. This effect provides a high-speed and energy-efficient tuning mechanism that is essential for advanced photonic systems. Two main types of EO effects are typically utilized in tunable photonic devices.

The Pockels effect, also known as the linear EO effect, occurs in non-centrosymmetric crystals such as LN [67] and BTO [68]. In these materials, the refractive index changes linearly with the applied electric field. The Pockels effect enables fast modulation speeds, with response times in the gigahertz range, and is commonly employed in modulators, tunable filters, and phase shifters. One of the key advantages of LN lies in its intrinsic anisotropy. The material exhibits direction-dependent electro-optic coefficients, particularly the high r_33_ component, which can be exploited through a proper crystal orientation and waveguide design to achieve efficient modulation. This anisotropy enables the optimization of modulator performance, making LN highly suitable for high-speed and low-drive-voltage applications [69]. LN modulators are well-established in optical communications due to their high performance and reliability [70]. The Kerr effect, or the quadratic EO effect, is present in all materials and causes a refractive index to change that is proportional to the square of the applied electric field [71]. While generally weaker than the Pockels effect, the Kerr effect is useful in nonlinear optics and specific high-field applications [58].

Electro-absorption effects are another category of EO modulation, involving electric-field-induced changes in the material’s absorption properties [72]. The Franz Keldysh effect in bulk semiconductors and the quantum confined Stark effect in quantum wells are two examples widely used in electro-absorption modulators [73,74]. These devices are particularly valuable in compact, high-speed optical interconnects based on III-V semiconductors. Emerging materials such as indium tin oxide (ITO) [75] and transparent conducting oxides (TCOs) [76,77,78] offer significant EO tunability. These materials support strong field-induced changes in permittivity and are being explored for integration into nanoscale and hybrid Si plasmonic devices, enabling high-performance modulation with a minimal footprint.

**Figure 2:**
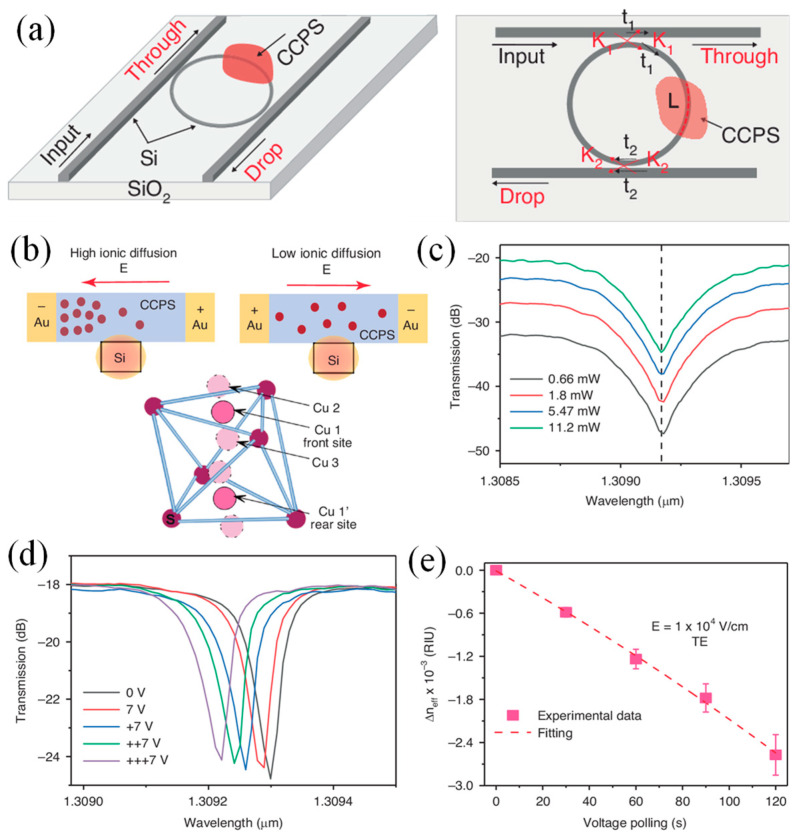
(**a**) Three-dimensional and cross-sectional views of the Si-MRR design incorporating CCPS. (**b**) A diagram illustrating the redistribution of copper ions within the CCPS/Si heterostructure when an electric field is applied, highlighting the movement of copper ions inside the sulfur cage. (**c**) Transmission spectra of the MRR measured at different input optical powers. (**d**) Transmission spectra of the MRR at a fixed voltage of 7 V over increasing polling times. (**e**) Change in the effective refractive index as a function of polling time, with an applied electric field of 1 × 10^4^ V/cm [36].

Two-dimensional materials like transition metal dichalcogenides (TMDs) and graphene have shown impressive optical responses to external stimuli, but challenges remain in achieving efficient modulation in the short-wave infrared (SWIR) region. Maintaining precise phase control and minimizing signal loss within a compact form factor is particularly difficult. Dushaq et al. investigated the electro-refractive behavior of multilayer CuCrP_2_S_6_ (CCPS) in the near-infrared wavelength range [36]. By embedding CCPS into Si photonic MRRs, the light–matter interaction was enhanced, allowing for improved sensitivity to phase and absorption changes (Figure 2a). The application of an electric field enabled the tuning of the n_eff_ by about 2.8 × 10^−3^ RIU, with minimal impact on extinction ratios and the resonance linewidth. The devices also display low optical loss and high modulation efficiency of 0.25 V·cm, with a consistent blue shift in resonance wavelengths regardless of the voltage polarity.

Figure 2b illustrates the redistribution of Cu ions in the CCPS/Si heterostructure under an electric field, altering both the copper ion concentration and electrical conductivity. The soft Cu–S bond allows Cu ion movement within and across the van der Waals gap, enabling optical tuning controlled by voltage. Figure 2c shows the transmission spectra of the Si/CCPS chip at varying laser input powers (0.66 mW to 11.2 mW) with a 0 V bias, revealing no significant resonance peak shifts, indicating no thermal dissipation. Measurements were limited to 11.2 mW to prevent power variations from affecting the results. Figure 2d presents the transmission spectra at bias voltages of 0 V and 7 V for TE polarization. With a constant 7 V bias, a consistent blue shift in resonance wavelengths is observed, indicating electro-optical modulation. The application of bias does not affect extinction ratios or linewidths, suggesting that Cu ion migration does not influence the imaginary refractive index. Figure 2e shows the change in n_eff_ as a function of polling time at 7 V, with a tuning efficiency of −8.3 pm/V. The n_eff_ can be tuned by 2.8 × 10^−3^ RIU, and the half-wave voltage-length product is estimated to be 0.25 ± 0.07 V·cm. Pre-poling of the CCPS material accelerates ion migration, like behaviors observed in ferroelectric PZT materials. Distinct differences in EO tuning are observed between transverse electric (TE) and transverse magnetic (TM) modes, indicating a polarization-dependent response. This characteristic expands the potential applications for light manipulation. The combination of optoelectronic and ionotronic properties in two-terminal CCPS devices offers significant promise for a wide range of applications, including optical switching, phased arrays, environmental sensing, optical imaging, and neuromorphic systems such as light-sensitive artificial synapses [36].

## 3. Key Materials for TO Applications

TO tunable photonic devices play a vital role in modern optical systems, offering a reliable and power-efficient means of controlling light without requiring mechanical movement or complex electronic modulation. Key components such as switches [79], modulators [80], filters [81], and phase shifters [82,83] have been developed using TO principles, particularly on platforms like Si and LN, where strong TOCs enable efficient tuning. Their significance lies in their ability to provide compact, low-cost, and complementary metal-oxide semiconductor (CMOS)-compatible solutions for dynamic optical signal processing, wavelength routing, and reconfigurable photonic networks, making them essential building blocks in applications ranging from data centers and telecommunications to quantum photonics and lab-on-chip systems [33].

Key materials used in TO applications are chosen for their strong temperature-dependent refractive index, optical transparency, thermal stability, and compatibility with photonic integration (Table 1). Si is a widely adopted material due to its high TOC, efficient light confinement, and compatibility with standard CMOS fabrication processes [80,84]. Polymers are also frequently used because of their customizable properties and significant thermal response [85]. Silica, while having a lower TOC, is valued for its low optical loss and excellent thermal and chemical stability [86,87]. Other advanced materials include chalcogenide glasses, known for their high refractive index modulation [62], and PCMs such as germanium antimony telluride, which allow reversible and energy-efficient tuning [88]. The selection of material depends on factors such as the desired modulation speed, power efficiency, scalability, and the specific requirements of applications like optical switching, sensing, and reconfigurable photonic circuits.

**Table 1:** Key characteristics of TO materials.

Material	Key Characteristics	Typical Wavelength Range	Advantages	Challenges
Silicon [60]	High TOC, widely used in integrated photonics, CMOS-compatible	1200 nm to 1700 nm (NIR)	Well-established fabrication process, cost-effective, easy integration with existing semiconductor tech	Low TOC compared to other materials (polymers or chalcogenides), sensitivity to temperature fluctuations, and a limited wavelength range
Silicon nitride [89]	High thermal stability, low loss in the visible to near-infrared range, relatively high TOC	400 nm to 1600 nm (VIS-NIR)	Low-loss, high-performance for visible and near-infrared, compatible with standard photonic integration	Higher fabrication complexity, less widely used in industry compared to Si
Lithium niobate [81]	High EO and TOCs, high-index contrast	600 nm to 1600 nm	Strong TO effect, suitable for high-performance photonic devices, widely used for modulators	Expensive, difficult to integrate with Si, handling issues due to fragility
Silicon carbide (SiC) [90]	High thermal conductivity, wide bandgap, stable at high temperatures	800 nm to 2000 nm	Extremely high thermal stability, works well in harsh environments, good for high-power applications	Expensive, difficult to process and integrate with other materials, and not widely used in photonics
Polymer-based materials (e.g., PMMA, SU-8) [61]	Moderate TOCs, flexible, lower refractive index contrast	400 nm to 1550 nm	Cost-effective, flexible, easy to process, adaptable for low-loss devices, and integration with flexible substrates	Lower TOCs compared to inorganic materials, lower stability under temperature cycling
Gallium arsenide (GaAs) [91]	High TOC, widely used in optoelectronics	800 nm to 1700 nm	High performance in photonic devices, used for high-speed communications and optical switching	Difficult to integrate with Si, expensive, challenging to scale for large-scale photonic circuits
Chalcogenide glasses [62,92]	High refractive index, large TOCs, used in infrared photonics	1000 nm to 5000 nm (NIR-MIR)	Excellent performance in the infrared range, suitable for nonlinear optics and low-loss waveguides	Not CMOS-compatible, expensive, and difficult to manufacture on a large scale
Silica [93]	Very low TOC, wide transparency window, excellent optical quality	400 nm to 2500 nm	Low optical loss, highly CMOS-compatible, mature fabrication processes	Not actively tunable, poor thermal and electro-optic tuning, low refractive index contrast

A compact light modulator based on Si photonic crystal (PhC) technology was designed, fabricated, and experimentally validated for operation near the 1.55 µm wavelength under TE polarization [80]. The core mechanism relied on tuning the cutoff frequency of a line-defect PhC structure, which was sensitive to changes in the Si refractive index. This tuning was achieved through localized thermal modulation, exploiting Si’s strong TO effect. The PhC consisted of a triangular lattice of cylindrical air holes etched into an SOI platform. Optical measurements confirmed effective TO control of the cutoff frequency, demonstrating the device’s potential for wavelength-selective modulation [80].

**Figure 3:**
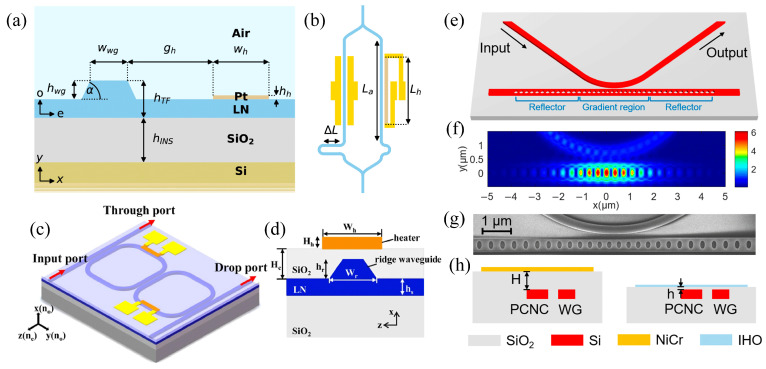
(**a**) Cross-sectional diagram of the TOPS, featuring the LNOI ridge waveguide and the platinum (Pt) heater layer [94]. (**b**) Asymmetric MZI configuration, where the first arm incorporates an EOPS and the second arm uses a TOPS, enabling a comparison between the two phase-shifter types [94]. (**c**) Schematic representation of the LNOI TO tunable optical filter [81]. (**d**) Cross-sectional view illustrating the waveguide and heater structure [81]. (**e**) Diagram illustrating the structure of the PCNC [97]. (**f**) Simulation of the electric field distribution within the PCNC at the resonant wavelength [97]. (**g**) SEM image showing the PCNC [97]. (**h**) Simplified cross-sectional view of the TO switch, with the waveguide (WG) labeled [97].

Maeder et al. explored the impact of geometry on the performance of metal strip TO phase shifters (TOPSs), both through modeling and experimental characterization [94]. To validate the simulated geometries, imbalanced Mach–Zehnder interferometers (MZIs) were fabricated on a 600 nm X-cut LNOI chip. The chip featured a 2 μm SiO_2_ insulation layer and a 500 μm Si handle. Ridge waveguides with a 1 μm width were formed by etching 200 nm of the LN layer, as illustrated in Figure 3a,b [94]. The results showed a 10-fold reduction in the voltage-length product compared to EO phase shifters (EOPSs), with bandwidths exceeding 100 kHz. Additionally, the footprint remained minimal, demonstrating the potential of TOPSs as efficient, small-scale building blocks for stable tuning and switching in LNOI photonic circuits.

Valley photonic crystals (VPCs) are optical materials that guide light using the valley degree of freedom. By breaking the inversion symmetry, they support valley-polarized edge states, enabling robust and directional light flow. VPCs are useful for advanced photonic devices and communication systems. VPCs have been investigated as an effective means to suppress backscattering and support robust light propagation through sharp bends, offering a viable route to realizing compact and low-loss components for integrated optical communication systems. However, limited research has been conducted on enabling power-efficient tunable functionalities in VPC-based devices, which are crucial for implementing essential operations such as optical switching and routing. Sun et al. proposed a thermally tunable add-drop filter (ADF) based on VPCs and experimentally verified at telecommunication wavelengths [95]. Topologically protected edge states and the negligible scattering associated with sharp bends were exploited to achieve a minimized device footprint of 17.4 × 28.2 μm^2^ and a low insertion loss of 2.7 dB. A diamond-shaped micro-loop resonator was introduced to confine optical modes and enhance thermal interaction through a microheater. As a result, a switching power of only 23.97 mW was required to redirect the output signal between ports. High-speed data transmission at a rate of 132 Gb/s was demonstrated using the thermally tuned ADF, with performance preserved under topological protection. These results have shown that thermally tunable VPCs based on Si can be utilized to develop reconfigurable, topologically protected photonic devices suitable for next-generation on-chip communication systems [95].

A thermo-optically tunable optical filter was designed and fabricated on a lithium niobate on insulator (LNOI) platform, which offers strong optical confinement while preserving the advantageous properties of LN [81]. The filter was implemented using two cascaded racetrack MRRs, a configuration selected to achieve high spectral performance (Figure 3c). As shown in Figure 3d, the ridge waveguide has a 260 nm etching depth, 1.2 µm width, and 72° sidewall angle for single-mode operation. A 1 µm top cladding isolates the MRRs from the heaters, minimizing optical loss. The micro-heater has a Ti thickness of 150 nm and a width of 2.5 µm.

A flat-top passband with an intra-band ripple of less than 0.3 dB was obtained, along with a 3 dB bandwidth of 4.8 GHz and an out-of-band rejection of approximately 35 dB. The total insertion loss was measured at around −14 dB, comprising roughly −6.5 dB from grating coupler losses and less than −1 dB from on-chip losses. Center wavelength shifts of over one free spectral range (FSR) were achieved using a heating power of approximately 89.4 mW. This device has been identified as a suitable candidate for use in optical signal processing and microwave photonic applications due to its tunability and high filtering performance [81].

Ultra-broadband, low-loss TO switches were recently demonstrated on a Si platform, employing a 2 × 2 MZI structure integrated with bent directional couplers [96]. These MZI-based switches were further scaled to N × N configurations and were integrated with arrayed waveguide gratings to realize compact reconfigurable add-drop multiplexers. To improve thermal tuning efficiency, a transparent graphene nano-heater directly contacting the Si core was experimentally implemented and validated. These advancements have established new possibilities for developing high-performance, thermally tunable PICs on Si [96].

A photonic crystal nanobeam cavity (PCNC) is a nanoscale optical structure that traps light by introducing a defect in a periodic nanobeam waveguide. It offers strong light confinement with a high-quality factor and small mode volume. These cavities are widely used in optical sensing, lasers, and quantum photonics. An on-chip TO switch was efficiently designed and validated through experiments, incorporating a PCNC in conjunction with a microheater made of hydrogen-doped indium oxide (IHO) [97]. The compact mode volume of the PCNC, along with the localized heating effect enabled by the transparent and conductive IHO, significantly boosts the interaction between thermal and optical fields, thereby enhancing the TO tuning performance. A top-view schematic of the device, shown in Figure 3e, features a waveguide patterned with 40 air holes arranged symmetrically. These holes define a central graded region flanked by two reflector sections. Each reflector, comprising 13 air holes, generates a photonic bandgap that confines light within the central cavity. This central region is engineered to minimize scattering and ensure efficient phase matching. For high-extinction-ratio (ER) operation, the widths of the nanobeam and its adjacent waveguide are set to 500 nm and 380 nm, respectively, with 225 nm spacing. Figure 3f illustrates the simulated resonant electric field distribution, highlighting that the optical field is concentrated in the 14-hole central zone, yielding an effective TO tuning length of approximately 4 μm [97].

Figure 3g presents an SEM image of the coupled waveguide and central gradient region. Figure 3h compares cross-sectional views of TO switches with conventional metal and IHO microheaters. The conventional switch uses a 100 nm NiCr alloy microheater with a 1 μm thick SiO_2_ layer, while the IHO microheater, made from a 70 nm-thick IHO film, reduces absorption loss. The SiO_2_ isolation layer is thinner (200 nm) to improve heat conduction without affecting optical resonance in the PCNC. Experimental results reveal a TO tuning efficiency of 1.326 nm/mW, with measured rise and fall times of 3.90 μs and 2.65 μs, respectively. The extinction ratios of the switches, measured at 25.8 dB and 27.6 dB, show that the IHO microheater introduces negligible insertion loss compared to conventional metal microheaters. This work demonstrates the strong potential of the TO switch as a critical unit for large-scale on-chip integrated arrays [97].

## 4. Key Material Classes for EO Applications

Key EO materials for tunable photonic devices are chosen based on their ability to efficiently change the refractive index in response to an electric field, enabling high-speed and low-power modulation (Table 2) [34,98]. LN is a leading material due to its strong linear EO effect, broad optical transparency, and stable performance, making it ideal for modulators and switches [25,70]. Gallium arsenide [99] and indium phosphide [100] are also widely used, especially in high-frequency applications, because of their high electro-optic coefficients (EOCs) and compatibility with active photonic components like lasers and detectors. EO polymers offer advantages such as low voltage operation, fast responses, and ease of processing, making them suitable for flexible and compact devices [101]. BTO provides very high EO activity and is being explored for integrated platforms, although it presents fabrication challenges [58]. Recent advances in thin-film LN have further improved integration, allowing for compact, efficient, and scalable devices [70]. The selection of the material depends on the specific requirements of the application, including the speed, footprint, integration method, and operating wavelength.

Lead zirconate titanate (PZT) is a ferroelectric ceramic material widely known for its strong piezoelectric and EO properties [102]. With electro-optic coefficients (e.g., r_33_) reaching up to ~400 pm/V in optimized thin-film configurations, PZT is a promising material for compact, low-voltage photonic modulators [103]. Its ability to be integrated onto silicon substrates also enables hybrid photonic platforms that leverage both EO and piezoelectric functionalities. However, challenges such as optical losses at telecom wavelengths, complex fabrication, and poling uniformity remain barriers to widespread adoption. Despite this, PZT continues to attract interest for applications in integrated optics, tunable devices, and multifunctional photonic systems [104,105].

**Figure 4:**
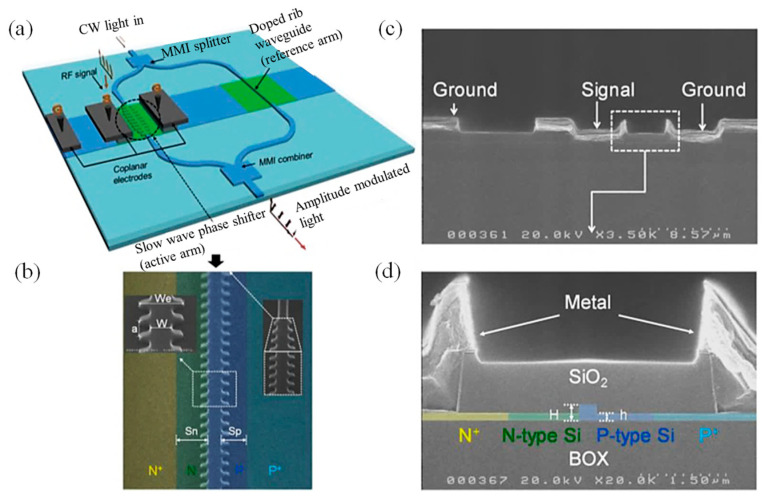
(**a**) Schematic of the modulator using an asymmetric MZI with MMI couplers for light splitting and combining. (**b**) Transverse SEM image showing the device layout and ground-signal-ground (GSG) coplanar electrodes. (**c**,**d**) Top and cross-sectional SEM views of the corrugated waveguide. The left inset highlights the taper for low-loss coupling between rib and slow-light sections; the right inset zooms into the corrugated structure. Doped regions are marked in color [56].

As global data traffic continues to grow, the demand for high-bandwidth, energy-efficient communication systems intensifies. While current optical networks effectively handle data transmission over long and medium distances, the need for low-cost, scalable short-reach interconnects has brought Si photonics to the forefront. Its potential for mass production and integration makes it a strong candidate for next-generation chip-scale optical systems. A key challenge in this space is achieving efficient electrical-to-optical signal conversion using modulators that are both compact and high-speed. In response, Brimont et al. [56] presented a Si-based EO modulator that combined slow-light enhancement in a nanostructured periodic waveguide with a high-speed semiconductor pn junction. Slow light was implemented using a 1D periodic waveguide with alternating narrow (W = 300 nm) and wide (We = 650 nm) segments, repeated every 310 nm (Figure 4a,b). The waveguide height was 220 nm, leaving a 100 nm-thick slab after partial etching (Figure 4c,d). Phase modulation was achieved via a reverse-biased pn junction formed near the waveguide core. Heavily doped p+ and n+ regions placed 500 nm (S_p_) and 550 nm (S_n_) from the waveguide edge were contacted using AlCu electrodes. Doping concentrations are 3 × 10^17^ cm^−3^ (p), 1.5 × 10^18^ cm^−3^ (n), and 1 × 10^20^ cm^−3^ (p+, n+). This synergistic design enabled a highly efficient, 500 µm-long modulator capable of error-free data transmission at 20 Gbit/s. Moreover, the device demonstrated the potential for modulation rates up to 40 Gbit/s, underscoring its suitability for future low-power, high-density photonic interconnects. These results established a promising pathway toward ultrafast, scalable networks-on-chip, enabling the next generation of high-performance computing systems [56].

Zhang et al. presented a novel strategy for achieving the significant miniaturization of these modulators by leveraging topological photonics [106]. This design utilized a one-dimensional topological interface state implemented on a thin-film LN integrated platform. This configuration enabled strong optical confinement and a pronounced EO enhancement at the topological interface. As a result, a topological cavity with a compact footprint of just 1.6 × 140 μm^2^ was realized, achieving an exceptional modulation bandwidth of 104 GHz. This represents the most compact LN-based modulator with a bandwidth exceeding 28 GHz. The device supported high-speed signal generation, including 100 Gb/s non-return-to-zero (NRZ) and 100 Gb/s four-level pulse amplitude modulation (PAM4). Owing to the reduced mode volume and low device capacitance, the modulator achieved an ultralow switching energy of 5.4 fJ/bit. By reducing the response time of topological photonic devices from the microsecond to the picosecond regime, this work established a foundation for scalable, high-speed LN PICs [106].

**Table 2:** Key characteristics of EO materials.

Material	Key Characteristics	Typical Wavelength Range	Advantages	Challenges
Lithium niobate [107,108]	Benchmark EO material: advances in thin-film and LNOI platforms	420 nm to 5200 nm	High EO efficiency; wide transparency window (0.4–5.5 µm); mature fabrication techniques	CMOS integration is challenging due to material incompatibility [25]
EO polymers [109]	High-speed modulation potential; organic materials with tunable properties	1310 nm to 1650 nm	Ultrafast response; potential for flexible integration	Stability issues under high temperatures; integration challenges with CMOS processes [101]
Barium titanate and Perovskites [110]	High EOCs (e.g., r_42_ ≈ 923 pm/V); emerging perovskite materials	500 nm to 1600 nm	Strong modulation performance; compatibility with CMOS platforms [110]	Material stability and uniform deposition remain a challenge
III–V Semiconductors (GaAs, InP) [111]	Active modulation with integrated electronics; wavelength-dependent performance	850 nm to 1650 nm	Direct bandgap materials enable efficient light emission; integration of active components like lasers and detectors	Limited CMOS compatibility; performance varies with wavelength [109]
Lead zirconate titanate [104,105]	Ferroelectric ceramic with high EOC (r_33_ up to ~400 pm/V); piezoelectric properties	1310 nm to 1650 nm	High EO efficiency; can be integrated as thin films on silicon; dual EO and piezoelectric functionality	Optical losses at telecom wavelengths; deposition and poling uniformity challenges

## 5. Device Architecture and Integration Platforms

The ability to manipulate light in photonic devices using EO and TO effects is pivotal in many modern applications, including telecommunications, sensing, and information processing [84,112]. The integration of various materials and the choice of platform for these devices significantly impact their performance, scalability, and potential applications. In this section, key considerations and innovations in device architectures and integration platforms are discussed, focusing on waveguides [41], modulators [25], switches [21], and tunable filters [113], as well as bulk vs. thin-film integration, heterogeneous integration, and the role of plasmonics [76] and metasurfaces (MSs) [114].

### 5.1. Waveguides, Modulators, Switches, and Tunable Filters

Waveguides are essential components in integrated photonics, guiding light through a structured medium [115,116]. Modulators exploit the EO effect to change the phase, amplitude, or frequency of the optical signal by applying an external electric field. These devices, often based on MZI [106,117,118], electro-absorption modulators (EAMs) [119,120,121], or micro-MRRs [122,123], enable high-speed optical communication systems. Switches, on the other hand, utilize the modulation of light to enable the routing of optical signals, and tunable filters offer wavelength-selective filtering, which is particularly useful for wavelength division multiplexing (WDM) in optical networks [36,124,125]. The integration of EO and TO tuning mechanisms into these devices significantly improves their versatility and response times.

Recent research has demonstrated that the displacement of thermally excited charge carriers under a strong electric field induces a second-order nonlinearity in silicon nitride (SiN), thereby enabling the linear EO effect despite the material’s inversion symmetry. Lafforgue et al. demonstrated an optically assisted poling for an SiN microring resonator, eliminating the requirement for high-temperature device processing [126]. The optical stimulation of charges was employed to circumvent the technical limitations associated with elevated temperatures. Through optimization of the poling process, a long-term effective second-order nonlinearity of 1.218 pm/V was obtained experimentally. Furthermore, the modulator’s high-speed EO response was measured, exhibiting a bandwidth of 4 GHz, which was limited by the microring resonator’s quality factor. This development advances the implementation of monolithic, compact SiN EO modulators, essential components for high-density integrated optical signal processing [126].

A 2 × 2 digital optical switch using two symmetrical unidirectional Bragg grating couplers was proposed by Sun et al. [127]. A low-loss polymer was used as waveguide material, and the Bragg grating coupling efficiency was optimized to be 22%; then, the unidirectional coupling efficiency of 99.9% was achieved in theory. The performance of the switch based on the unidirectional couplers with Bragg gratings was theoretically modeled and simulated. Finally, the 2.4 dB insertion loss, the −17 dB crosstalk between two output ports, the 28 dB extinction ratio, the 1.5 ms response speed, and the 87 mW power consumption were experimentally demonstrated with this regime [127].

**Figure 5:**
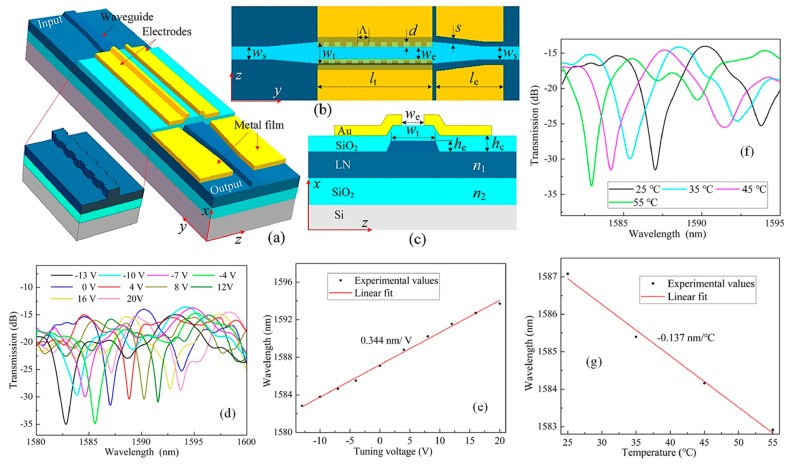
(**a**) Diagram of the LPWG filter; (**b**) top view layout, and (**c**) cross-section of the EO tuning area. The inset in (**a**) shows asymmetric gratings on the ridge sidewalls without the buffer layer and electrodes. (**d**) Transmission spectra at tuning voltages from −13 to 20 volts, and (**e**) center wavelength shift with voltage and linear fit. (**f**) Transmission spectra at temperatures from 25 to 55 degrees Celsius, and (**g**) center wavelength shift with temperature and linear fit [113].

Li et al. presented a compact, electro-optically tunable optical filter designed on an LNOI platform, utilizing sidewall long-period waveguide gratings (LPWGs) [113]. Figure 5a,b present the schematic representations of the device in three-dimensional and top views, respectively. The filter architecture incorporated two key components: corrugated sidewall LPWGs to enable mode coupling and metal ribbons for absorption, with a tapered waveguide facilitating the filtering process. This combination allowed for precise control over light propagation within the waveguide. Each grating structure on the sidewalls is defined by a common set of parameters. These include the grating period (Λ), the etch depth (d), a relative phase shift of one-half period between the two gratings, and a duty cycle of 50 percent. EO tuning is achieved using a pair of chromium and gold electrodes that were aligned along the length of the two-mode rib waveguide. These electrodes were separated from the waveguide by a silicon dioxide buffer layer with a thickness (h_c_), as shown in Figure 5c. The spacing between the electrodes is labeled as (w_e_), and their length is equal to that of the two-mode waveguide section.

Figure 5d shows the normalized transmission spectra measured at 25 degrees Celsius. As the voltage increases from 0 to 20 volts, the center wavelength redshifts from 1587.1 to 1593.7 nm, with the contrast decreasing from 16.32 to 13.68 decibels. When the voltage decreases to minus 13 volts, the center wavelength blueshifts to 1582.8 nm, with the contrast dropping to 11.66 decibels before rising to 15.22 decibels. Figure 5e confirms a near-linear shift in the center wavelength with a tuning rate of approximately 0.344 nm per volt, while the contrast variation remains nonlinear, as seen in Figure 5d. The thermal response of the packaged filter was examined by observing how the center wavelength of the rejection band changes with ambient temperature. Normalized transmission spectra were recorded at various temperatures ranging from 25 to 55 degrees Celsius, as shown in Figure 5f. As the temperature increased within this range, the center wavelength shifted to shorter values, moving from 1587.1 nm to 1582.9 nm. This blueshift followed an approximately linear trend, with a rate of about 0.137 nm/°C, as depicted in Figure 5g. Due to its compact form and compatibility with other LNOI-based components, this filter is well-suited for integration into more complex photonic circuits aimed at advanced on-chip optical signal processing [113].

### 5.2. Bulk vs. Thin-Film Integration

The integration of photonic devices can be approached through two primary strategies: bulk integration and thin-film integration. In the bulk integration approach, waveguides and other photonic components are fabricated on large substrates with relatively thick layers of material, such as LN or bulk crystals [35]. Bulk materials typically offer high nonlinearities and strong EO effects, making them suitable for high-performance modulators and switches [128]. However, the scalability of bulk devices in integrated photonics is limited by their size, weight, and fabrication complexity [98].

Thin-film integration, which involves the deposition of thin layers of materials onto substrates like Si, SiN, or glass has become more dominant in recent years [129,130,131]. Thin-film devices allow for greater miniaturization, reduced material usage, and easier integration with existing semiconductor fabrication technologies [132]. Materials like Si, SiN, and polymers are widely used for this purpose, as they provide a range of optical properties and enable compact, scalable photonic circuits [133]. Thin-film integration is particularly attractive for large-scale PICs, which can be fabricated using standard photolithographic techniques [134]. The trade-offs between these integration platforms often come down to factors like device performance, ease of integration, and cost. While bulk materials provide superior optical properties, thin-film devices offer flexibility and scalability suitable for high-density integration [133].

Thin-film lithium niobate (TFLN) EO modulators have emerged as leading candidates for advancing modern photonic systems. By leveraging the inherent strengths of LN, such as minimal optical loss, high signal extinction, excellent linearity, and strong optical power tolerance, these devices outperform traditional bulk modulators in key areas. In particular, TFLN modulators significantly enhance the voltage-to-bandwidth efficiency. Despite these advantages, their compact electrode spacing introduces considerable microwave attenuation due to metal-induced absorption, which restricts performance at higher frequencies [135].

**Figure 6:**
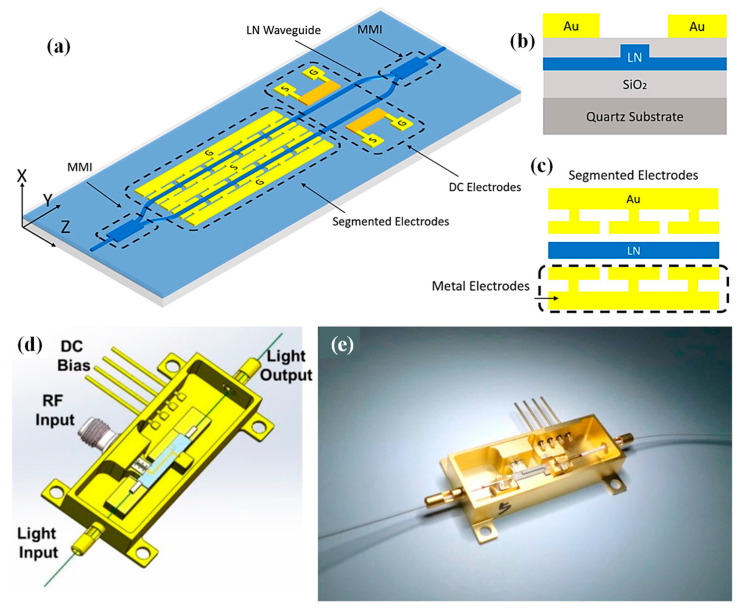
(**a**) Illustration of the LN modulator architecture, (**b**) detailed cross-sectional representation of the electrode segmentation and (**c**) overhead view highlighting the electrode arrangement. Internal configuration of the EO modulator package: (**d**) conceptual layout and (**e**) corresponding physical images [135].

To address this, Tao et al. engineered traveling-wave electrodes with precision microstructures, successfully extending the 3 dB EO bandwidth to 51.2 GHz [135]. Figure 6 presents the schematic of the nanophotonic LN modulator in panel (a), with the cross-sectional (b) and top (c) views of the electrode design shown in the following panels. The fabricated devices also demonstrated excellent performance metrics, including sub-2 dB optical insertion loss and a 15 dB extinction ratio. Encapsulated in metal packages, the modulators underwent and passed comprehensive reliability assessments. This development marked a pivotal milestone in bringing TFLN modulator technology closer to widespread application and commercial viability [135].

The modulator (Figure 6d,e) was housed in a 45 × 20 × 9 mm Kovar package, selected for thermal compatibility and sealed with nickel and gold plating. Inside, space was machined for the LN chip and microwave components. Optical feedthroughs use Kovar tubes. To reduce the coupling loss from mode mismatch, polarization-maintaining fibers with smaller cores and on-chip spot-size converters were used. Precision alignment within 0.1 μm and 0.05° was achieved using a six-axis platform. UV adhesive and solder secure the fibers, maintaining over a 20 dB polarization extinction. The actual coupling loss was around 20 dB. RF signals enter via 1.85 mm connectors rated to 65 GHz. A coplanar waveguide on an aluminum nitride substrate routes signals to the modulator, which is gold-wire bonded. Good grounding and 50 Ω termination resistors ensure low return loss. Seam welding seals the package, with helium leak testing confirming the integrity [135].

### 5.3. Heterogeneous Integration and Hybrid Material Systems

Heterogeneous integration refers to the combination of different materials or technologies to create a composite device with complementary properties [136,137]. In the context of tunable photonics, heterogeneous integration enables the use of a range of materials, such as Si for efficient waveguiding and EO materials like LN or III-V semiconductors for fast modulation [138,139]. This allows for devices that leverage the strengths of each material while mitigating individual limitations [140,141].

For example, the hybrid integration of Si photonics with LN offers an attractive path for developing high-performance modulators and switches that can operate at high speeds, while still maintaining the compactness and scalability of Si [142,143]. This integration can also facilitates the combination of passive waveguides with active modulation regions, allowing for high-density photonic circuits with reduced power consumption. III-V semiconductor materials, such as indium phosphide (InP) and gallium arsenide (GaAs), can be integrated with Si to provide active devices like lasers and modulators, further enhancing the capabilities of photonic circuits [144,145,146,147].

Moreover, hybrid material systems can incorporate novel 2D materials such as graphene or transition metal dichalcogenides (TMDs), offering unique optoelectronic properties that are leveraged in tunable photonic devices [148,149,150]. These materials enable lower-power modulation, greater integration flexibility, and new functionalities, including tunable absorption, phase shifting, and enhanced light–matter interactions [151,152,153].

Advances in precision measurement technologies such as atomic clocks, inertial navigation systems, and experiments probing the foundations of physics have led to increasingly stringent demands on the laser frequency noise. Rubidium-based quantum systems require highly stable 780-nanometer lasers for optimal performance in timing, sensing, and quantum information processing. While these systems currently rely on discrete, tabletop laser setups, achieving wafer-level integration is essential for enabling compact and scalable photonic systems suitable for integration into chip-scale platforms.

Despite ongoing efforts to develop integrated 780-nanometer lasers with extremely narrow linewidths, achieving both a fundamental linewidth below one hertz and an integral linewidth under one kilohertz has remained a difficult goal. Isichenko et al. presented a hybrid integrated laser operating at 780 nm that demonstrates significant progress toward overcoming this challenge [154]. The laser utilized a self-injection locking technique and achieved a fundamental linewidth of 0.74 hertz and an integral linewidth of 864 hertz. Additionally, the laser reaches a thermorefractive noise floor of 100 hertz squared per hertz at a 10-kilohertz offset, representing over an order of magnitude improvement compared to prior integrated devices operating at the same wavelength.

**Figure 7:**
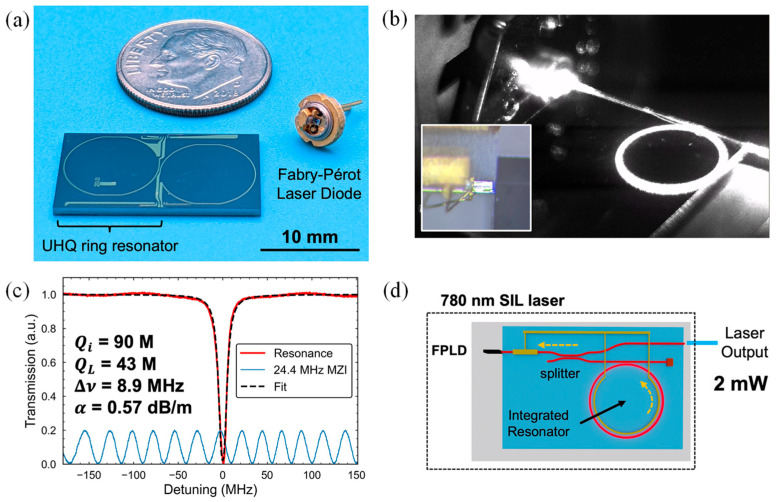
Hybrid-Integrated 780 nm self-injection locked (SIL) laser: (**a**) image of the chip-scale laser system, featuring an ultra-high-Q MRR and a Fabry-Pérot laser diode (FPLD), shown with a dime for scale, and (**b**) laser output locked to the resonator. The inset shows angled edge coupling of the FPLD. (**c**) Resonator transmission spectrum showing a loaded Q of 43 million, intrinsic Q of 90 million, 0.57 dB/m loss, and 8.9 MHz linewidth. Frequency calibration is performed using an unbalanced MZI (blue trace). (**d**) Schematic of the self-injection locked laser: the FPLD is edge-coupled to a chip with a splitter and a thermally tunable high-Q resonator. Output is collected through an edge-coupled cleaved fiber [154].

The chip-scale self-injection locked (SIL) laser system integrated a commercial 780 nm Fabry-Pérot laser diode (FPLD) with an ultra-high-Q SiN photonic chip. The FPLD, supplied in a TO-can package, was modified by removing its lid and is edge-coupled to the chip (Figure 7a,b). To align with the TM waveguide mode selected for its lower propagation loss, the diode was rotated by 90 degrees during coupling. The integrated chip comprised a high-Q MRR, a power splitter, and a thermal phase tuner. All components were fabricated using a CMOS-compatible SiN process. Near-critical coupling between the resonator and waveguide achieved a 20 dB extinction ratio. Combined with an intrinsic Q-factor of 90 million, this facilitates strong resonant Rayleigh backscattering, which provides narrowband optical feedback to the laser. Using an unbalanced MZI, the resonator was measured to have an intrinsic Q of 90 million and a loaded Q of 43 million. These values correspond to a 0.57 dB/m propagation loss and a linewidth of 8.9 MHz (Figure 7c). The resonator had a 5 mm radius, yielding a free spectral range (FSR) of 6.43 GHz.

The system supported two configurations: one optimized using a multi-axis alignment stage, and another in which the laser was permanently bonded to the chip in a hybrid package. Optical feedback from the resonator was strong enough to allow efficient power extraction via an on-chip directional coupler, delivering 2 mW of output power (Figure 7d). This output was over ten times higher than earlier chip-scale SIL lasers with a comparable frequency noise performance. To enhance coupling, the waveguide width was tapered from 4 μm near the resonator to 2 μm at the chip edge. The measured coupling loss between the FPLD and the chip was approximately 4 dB. The demonstrated performance illustrates the potential of this laser architecture for enabling compact and low-noise light sources suitable for a wide range of quantum sensing, computation, and metrology applications. Furthermore, the design principles can be extended to other atomic transition wavelengths, opening new opportunities for integrated quantum systems [154].

### 5.4. Role of Plasmonics and MS in Enhancing Effects

Plasmonics and MS have emerged as powerful tools in the field of photonics, providing new avenues for enhancing the EO and TO effects [114]. Plasmonics exploits the interaction between electromagnetic waves and free electrons in metal nanostructures to concentrate light into subwavelength volumes [155]. This can lead to enhanced light–matter interactions, particularly in miniaturized devices such as modulators and sensors [156,157,158]. In tunable photonic devices, plasmonic structures can be used to achieve stronger EO and TO effects by amplifying the local electric field or heat generation in the vicinity of the metal structures [159]. This allows for more efficient modulation and switching, with the potential for ultra-fast response times. Plasmonic modulators, for instance, exploit the strong field confinement of surface plasmon polaritons to enable the high-speed, low-power modulation of light [76,159].

MSs, which are engineered 2D materials with subwavelength patterns, offer another promising approach for enhancing tunable photonic devices [160,161,162]. MSs can control the phase, polarization, and intensity of light on demand, making them ideal for applications in beam shaping, tunable filters, and dynamic holography [8,163,164,165]. By carefully designing MSs with EO or TO materials, it is possible to achieve high-performance, low-loss tunable devices that offer both structural and material flexibility. In some designs, the combination of EO materials with MSs has led to devices that can dynamically control light propagation through induced changes in both phase and amplitude, creating tunable filters and modulators with low energy consumption [166,167,168,169].

**Figure 8:**
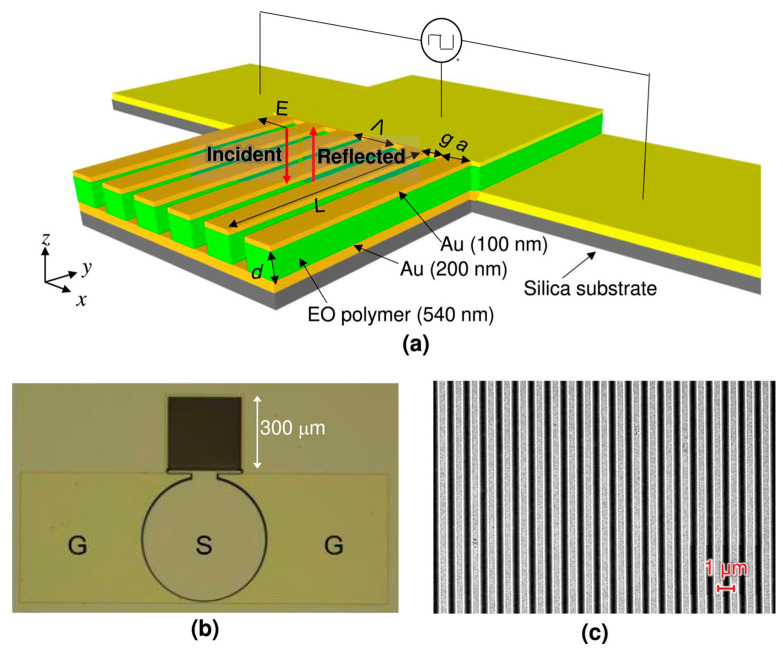
Overview of the MS modulator configuration: (**a**) a schematic illustration presents the overall structure of the device, (**b**) an optical microscope image captures the top view of the fabricated MS, and (**c**) a top-down SEM image reveals the detailed features of the grating, which has a periodicity of 1080 nm [170].

Zhang et al. experimentally demonstrated a high-speed EO modulator based on a plasmonic MS, designed to function in the near-infrared spectral region with gigahertz-level modulation bandwidth (Figure 8a) [170]. To enable the effective intensity modulation of reflected light, a carefully engineered subwavelength grating composed of alternating metal and insulator layers was employed. This structure supported bimodal plasmonic resonances, which were tuned to meet the condition for critical coupling. As a result, the device achieved near-total light absorption, measured at 99.8% or −27 dB, and exhibited a high-quality resonance with a Q-factor (λ_res_/FWHM) of 113 at a wavelength of 1650 nm [170].

Figure 8b,c display the top-view optical image and the SEM image of the fabricated device featuring a grating period of 1080 nm. For testing and measurement purposes, the device was wire-bonded onto a printed circuit board (PCB). The integration of an EO polymer within the grating enables a modulation depth of up to 9.5 decibels when a voltage of ±30 volts is applied. The modulator demonstrated a 3-decibel bandwidth of 1.25 gigahertz, with performance primarily limited by contact resistance and the output impedance of the driving circuit. Due to the high electrical conductivity of the metallic components and the compact structure that minimizes parasitic capacitance, the device holds strong potential for operation at frequencies well above the current bandwidth. This advancement opens new possibilities for ultrafast active MSs in a wide range of optical and photonic applications [170].

## 6. Performance Metrics and Comparison

The performance of tunable photonic devices based on EO and TO materials is assessed using a range of key metrics that determine their efficiency, practicality, and long-term viability in real-world applications. These metrics include the tuning efficiency, insertion loss, modulation bandwidth and speed, power consumption, and long-term stability.

### 6.1. Tuning Efficiency

Tuning efficiency quantifies how effectively a device can alter its resonant or operational wavelength in response to external stimuli, such as temperature or voltage [17,171,172]. It is typically expressed as the change in wavelength per unit temperature (dλ/dT) for TO materials or per unit voltage (dλ/dV) for EO materials. TO materials often exhibit higher tuning efficiency due to the relatively large TOC of certain polymers and semiconductors [5,173]. For example, Si has a TOC of approximately 1.86 × 10^−4^ per Kelvin, resulting in a substantial shift in resonance with moderate temperature changes. In addition, Chen et al. proposed a thermally tunable nanobeam cavity made of SiN, embedded within a polymer [173]. This device exhibits a tuning efficiency of 44 pm/°C and 0.13 nm/mW in the near-visible spectrum. The impressive tuning performance is driven by two factors: the high TOC of the SU-8 polymer and the unique “air-mode” cavity design, which allows a significant portion of the cavity’s optical field to be confined within the polymer region. This resonator offers a promising platform for localized tuning in cavity quantum electrodynamics experiments based on SiN [173]. Furthermore, EO materials such as LN demonstrate faster but often smaller shifts in wavelength per unit voltage due to the lower index modulation achievable via the Pockels effect [25]. The choice between these two mechanisms depends on the specific requirements for speed versus the tuning range.

**Figure 9:**
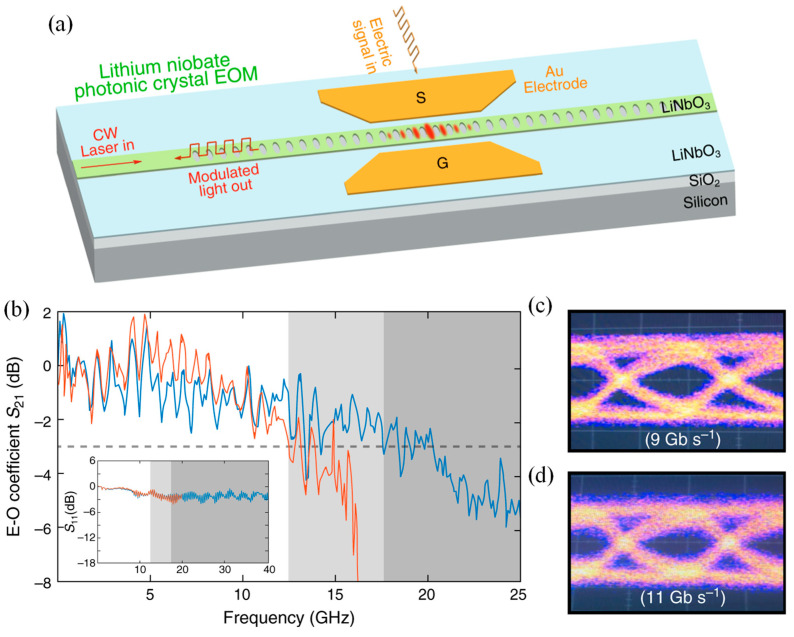
(**a**) Diagram of the LN photonic-crystal EO modulator. (**b**) Scattering parameter S21 for devices with optical Qs of ~14,000 (blue) and ~20,000 (orange). The gray regions represent the 3 dB bandwidth limits, and the dashed line indicates the 3 dB S_21_ limit. Inset: S_11_ reflection parameter for both devices. (**c**,**d**) Eye diagrams of the photonic-crystal EOM output, measured with a 2^7^−1 NRZ PRBS and V_pp_ = 2 V, with the laser wavelength locked at the cavity resonance [25].

Li et al. presented high-speed LN EO modulators built using photonic crystal nanobeam resonators (Figure 9a) [25]. These devices offered strong performance characteristics, including a high tuning efficiency of 1.98 GHz/V, a wide 17.5 GHz modulation bandwidth, and a minimal EO mode volume of just 0.58 μm^3^. The modulators efficiently drive high-Q cavity modes across both adiabatic and non-adiabatic regimes. Figure 9b shows the modulation response of one of the devices (blue curve), revealing a 3 dB bandwidth of approximately 17.5 GHz. This bandwidth is primarily constrained by the photon lifetime of the EOM cavity (~11 ps), as the electrode circuit’s broader spectral response is evident from the flat S_11_ reflection spectrum shown in Figure 9b.

Since the modulation bandwidth was mainly determined by the device’s optical Q, it can be easily adjusted for various applications by selecting devices with the desired optical Q. For comparison, the orange curve in Figure 9b illustrates a device with an optical Q of 20,000, which achieves a 3 dB bandwidth of around 12.5 GHz. The wide modulation bandwidth of these devices enables high-speed EO switching. To demonstrate this, an NRZ signal was applied with a (2^7^−1)-bit pseudo-random binary sequence (PRBS) to an EOM with a V_pp_ of 2.0 V. The resulting eye diagrams at two-bit rates, 9 and 11 Gb/s, are shown in Figure 9c and Figure 9d, respectively. Both diagrams exhibit clear eye openings, indicating successful high-speed operation. The demonstrated bit rates were limited by the maximum bit rate of the PRBS generator (Agilent 70843B), which caps at 12 Gb/s. However, the minimal degradation observed between Figure 9c,d suggests that the EOM could support even higher bit rates. This work lays the groundwork for large-scale LN-based photonic circuits, supporting emerging applications in data communication, RF photonics, and quantum information systems [25].

### 6.2. Insertion Loss, Bandwidth, and Speed

Insertion loss refers to the amount of optical power lost as light propagates through the tunable device [174,175]. Lower insertion loss is critical for high-performance photonic circuits to maintain signal integrity and minimize amplification requirements. EO modulators generally offer lower insertion loss compared to TO counterparts, largely due to the absence of heat-induced material absorption and scattering [176,177]. A new microheater design was introduced for efficient TO modulation in Si photonic platforms, utilizing indium tin oxide (ITO), a transparent, conductive material compatible with standard CMOS processes [178]. To induce a phase shift, a metallic heater was positioned above the Si waveguide, leveraging both Joule heating and the thermo-optic effect of Si. Since metals exhibit significant optical losses in the near-infrared range, an upper cladding layer was introduced to provide optical isolation between the heater and the waveguide. Nonetheless, using a thick cladding layer can negatively impact the phase shifter’s performance by increasing power consumption or reducing the switching speed. The design prioritized reducing the separation between the heater and waveguide to minimize optical losses. Performance analysis was carried out using finite element simulations, where ITO-based heaters were compared to traditional titanium heaters under different cladding conditions and for both TE and TM optical modes. Based on the simulation outcomes, ITO microheaters were fabricated with optimized structural parameters. Experimental validation showed that a heater with a length of 50 µm achieved a π phase shift using only 10 mW of electrical power and demonstrated response times in the microsecond range. These findings underscore ITO’s effectiveness as a low-loss, high-performance heating element and highlight its potential for supporting the integration of dense phase shifter arrays in future PICs [178].

The bandwidth and speed are essential parameters for dynamic modulation and switching applications [179,180,181,182]. EO devices typically offer superior speed, often reaching tens of gigahertz due to the instantaneous nature of electric field modulation. In contrast, TO devices are inherently slower, with modulation speeds generally limited to the kilohertz range because of the thermal diffusion processes involved. However, their simplicity and ease of integration can offset these speed limitations in applications that do not require rapid reconfiguration.

**Figure 10:**
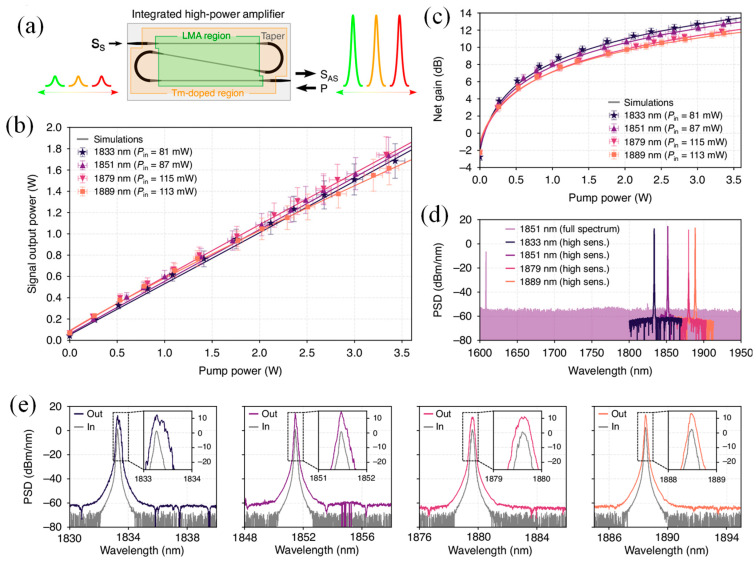
(**a**) Diagram of the counterpropagating setup where the pump enters from the opposite side of the seed laser. The seed is amplified using a large mode area amplifier. (**b**) Amplified output power as a function of pump input for seed wavelengths from 1830 to 1890 nm, with input powers between 81 and 115 milliwatts. (**c**) Net gain versus pump power, with error bars reflecting a 0.3 decibel coupling uncertainty. (**d**) Broadband output spectra show minimal ASE between pump and signal due to strong seed input. (**e**) Input and amplified output spectra across 1830 to 1890 nm, with observed gains of 10 to 12 decibels [183].

High-power tunable lasers are essential for applications such as telecommunications, sensing, and ranging. Integrated photonics has faced challenges in this area due to limited energy storage and low output power resulting from compact device sizes. In fiber optics, the introduction of large-mode-area fibers significantly improved power handling by expanding the optical mode, a concept that can also be applied at the chip level. Singh et al. introduced a Si photonics-based large mode area amplifier that enables a compact, high-power tunable laser [183]. The system delivered up to 1.8 watts of output across a tuning range of 60 nm, from 1.83 to 1.89 µm, constrained only by the seed laser source. Figure 10a illustrates the experimental setup, where pump and seed lasers were coupled into the chip using a custom-built laser source tunable from 1830 to 1890 nm. Coupling losses were approximately 2.2 decibels for the pump and 2.4 decibels for the signal. Both copropagating and counterpropagating configurations yielded similar results, as the strong seed signal suppressed amplified spontaneous emission. Polarization remained consistent throughout the chip, with an extinction ratio exceeding 30 decibels. A six-centimeter device length provided the best performance. Figure 10b–e show the amplified output power ranging from 1650 to 1800 milliwatts, achieving gains between 11.5 and 13.5 decibels. Even higher gains were observed but were limited by facet reflection, which can be minimized through index matching or angled facets in future integrated designs.

Figure 10d presents the broad output spectrum extending to 1950 nm, free from the amplified spontaneous emission background. Figure 10e compares the input and output spectra, showing a high signal-to-noise ratio and minor broadening due to back-reflection, which can be addressed with proper output isolation. Longer devices of 11 cm to 15 cm showed slightly reduced gains at current pump levels but are expected to perform better under increased pump power. The system maintained stable operation over extended durations. This approach provided a significant power boost over conventional integrated tunable lasers, which typically operate in the milliwatt range. The results show performance comparable to or better than many benchtop systems, indicating strong potential for integrated high-power photonic solutions in practical applications [183].

### 6.3. Power Consumption

Power efficiency is another critical factor, particularly for large-scale PICs and portable devices [184,185,186]. TO tuning generally requires continuous power input to maintain elevated temperatures, resulting in higher steady-state power consumption. EO tuning, on the other hand, can be more energy-efficient since the voltage application does not necessarily involve power dissipation if designed with capacitive actuation. For instance, Zhong et al. presented an Si EO switch based on photonic crystal nanobeam cavities, fabricated on a foundry-compatible platform [187]. The device featured ultra-low static tuning power (0.10 mW), low dynamic energy consumption (6.34 fJ/bit), and a compact footprint of 18 μm × 200 μm. High-speed performance was validated using a 136 Gb/s PAM-4 signal. This switch offered the lowest static power and highest data rate among its class, making it ideal for applications in optical computing, data center interconnects, neural networks, and reconfigurable photonics [187]. Nonetheless, the overall power budget also depends on the drive circuitry, device geometry, and system-level thermal management strategies [185].

**Figure 11:**
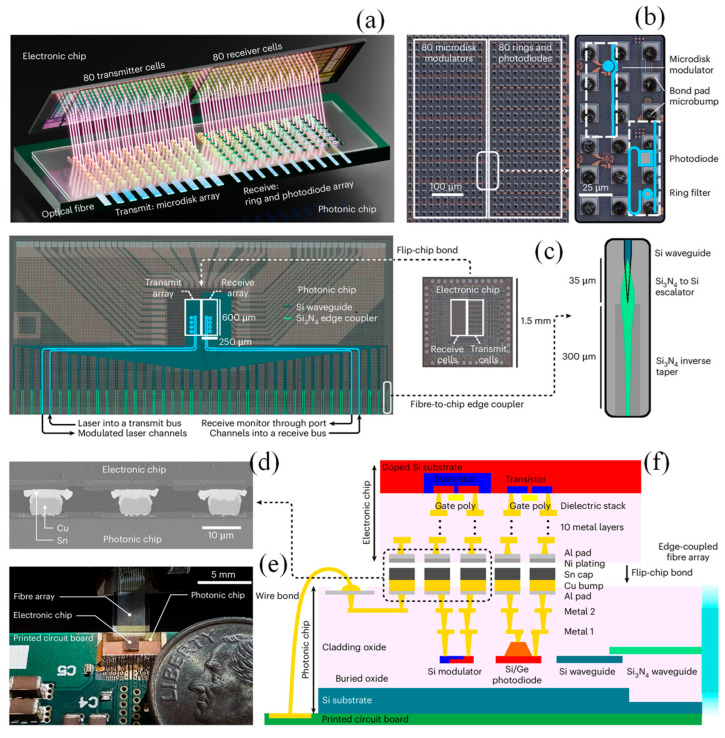
(**a**) Conceptual diagram of a vertically integrated system, aligning arrays of electronic circuits with corresponding photonic components. (**b**) Optical microscope image showing the 80-channel photonic array; the inset highlights a pair of transmitter and receiver units. (**c**) Chip-level images of the photonic and electronic dies. The central, white-marked area contains active photonic circuits, while the surrounding region distributes optical and electrical connections for fiber and wire interfaces. A blue trace indicates a sample four-channel waveguide route. Inset: schematic of a fiber-to-chip coupler featuring an SiN taper transitioning into Si. (**d**) SEM cross-section capturing the bonded interface between the electronic and photonic layers. (**e**) Assembled transceiver module mounted on a printed circuit board and aligned to a fiber array, with a U.S. dime shown for a size reference. (**f**) Layered view illustrating the chip stack materials, including Si bases, doped device layers, and metal interconnects for both the photonic and electronic components [184].

Scaling artificial intelligence (AI) systems requires not only powerful computation but also efficient communication between distributed processing units. A significant bottleneck in this process is the high energy cost and chip area needed for transferring data across chips. To overcome this limitation, Daudlin et al. presented a novel solution based on the dense three-dimensional (3D) integration of photonic and electronic components [184]. The photonic–electronic integration process for chip-to-chip communication is illustrated in Figure 11, which outlines each stage of the assembly in detail. The process begins with the formation of copper pillar bumps on the photonic chip. These structures are created through electroplating, where copper pedestals are topped with a thin tin layer. To complete the integration, the photonic chip was aligned with a nickel-coated electronic chip and bonded using heat and pressure (Figure 11a). Following this, the result of the microbump fabrication is shown in the microscope image of the photonic device array (Figure 11b). This array is embedded within a test photonic chip, which interfaces with a corresponding electronic test chip. Figure 11c presents this setup alongside a schematic depicting how optical fibers are coupled with the chip [184].

The bonding design includes 2304 interconnects laid out with a tight pitch, 15 μm spacing, and a 10 μm bump diameter, yielding a 25 μm pitch. This high-density layout required precise control to prevent issues such as tin overflow, which can lead to electrical shorts, and tin deficiency, which may weaken bond reliability. To evaluate the structural integrity of the bonds, cross-sectional SEM was used (Figure 11d). The images confirm successful isolation between bonds, and mechanical testing shows that the bonded chips withstand a separation force of 2.1 kg (114.9 MPa). Electrical modeling indicated that each bond exhibits a low capacitance of just 10 fF. Figure 11e showcases the complete transceiver assembly, which was mounted on a printed circuit board using wire bonds and aligned optically with a fiber array. A detailed layer-by-layer cross-section is shown in Figure 11f, illustrating the composition of the electronic and photonic chips, the bonding interface, and how connections are made to both the fiber and the board.

This configuration delivered a significantly higher density of 3D-integrated channels than previously demonstrated, enabling a total bandwidth of 800 Gb/s and an impressive spatial data rate of 5.3 Tb/s per mm^2^. The system also achieved exceptional energy efficiency, with the transmitter and receiver front ends consuming only 50 femtojoules and 70 femtojoules per bit, respectively, while operating at 10 Gb/s per channel. Such performance was made possible through a design that was fully compatible with standard 300 mm CMOS foundry processes, ensuring scalability and manufacturability. By minimizing the energy and area required for inter-chip communication, this platform offers a practical and scalable pathway to eliminate bandwidth limitations in next-generation AI hardware [184].

### 6.4. Long-Term Stability and Fatigue

Stability over time and resistance to fatigue are vital for ensuring the reliability of tunable photonic devices [188]. TO devices may suffer from material degradation and performance drift due to repeated heating and cooling cycles, which can affect polymer-based materials more significantly than crystalline semiconductors. EO materials such as LN and BTO exhibit better long-term EO stability and lower susceptibility to fatigue, though dielectric breakdown and charge trapping can pose reliability challenges if not properly mitigated. For both material classes, encapsulation techniques, thermal isolation structures, and robust packaging are essential to enhance the operational longevity [189].

A novel optoelectronic oscillator (OEO) architecture based on stimulated Brillouin scattering was proposed, offering both high-frequency stability and fine tunability [190]. Central to this design is the use of a single laser source to generate both the Brillouin pump and the optical carrier within the OEO loop. This shared-source approach ensures excellent coherence and stability in the resulting microwave signal. Fine frequency control was realized through an EO frequency shifter, which integrates a dual-parallel Mach–Zehnder modulator (DPMZM) with an electrical 90° hybrid coupler. By precisely adjusting the frequency of a low-frequency microwave signal applied to the DPMZM, the system enabled the generation of a high-frequency microwave output with fine resolution. Experimental validation demonstrated a continuous tuning range from 10.645 GHz to 12.645 GHz, achieved with a 10 MHz resolution. At a representative frequency of 11.145 GHz, the system exhibited minimal drift, with measured frequency and power fluctuations limited to 6.25 kHz and 0.77 dB, respectively, over a 1000 s interval. Moreover, by incorporating broadband optical and electrical components, the proposed OEO configuration was scalable and capable of supporting stable microwave signal generation up to 40 GHz or higher, making it suitable for a wide range of high-frequency applications [190].

EO polymer modulators with high thermal stability are increasingly in demand for advanced photonic applications. In response, Miura et al. developed a series of EO polymers exhibiting glass transition temperatures as high as 194 °C [188]. These materials were integrated into MZI modulators, which demonstrated exceptional thermal endurance. Specifically, the modulators maintained stable half-wave voltage performance during continuous operation at 105 °C for approximately 2000 h. To further evaluate their performance under thermal stress, the modulators were equipped with traveling-wave electrodes and tested across the 10–40 GHz frequency range as the operating temperature increased. The results revealed a consistently reliable frequency response, with modulation capabilities sustained up to 130 °C. These findings confirm the effectiveness of the developed EO polymers in supporting high-frequency, thermally robust modulators suitable for demanding environments [188].

**Figure 12:**
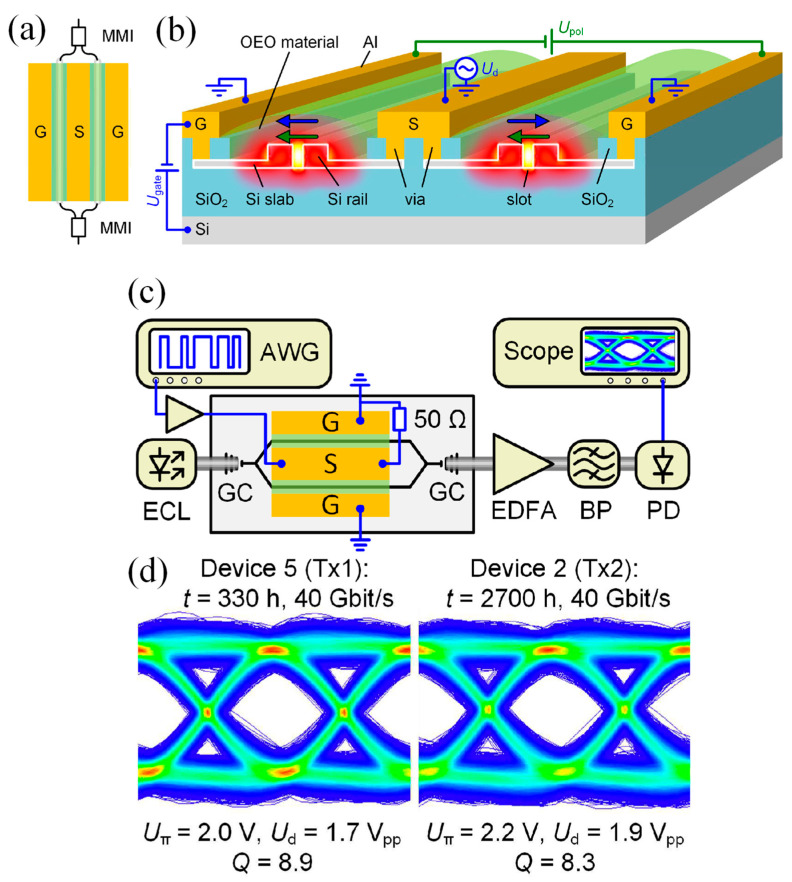
SOH MZM and data transmission: (**a**) top view of the SOH modulator showing the ground-signal-ground transmission line and multi-mode interference couplers. (**b**) Perspective view illustrating Si slot waveguides filled with EO material, with strong RF and optical mode overlap in the slot. EO activity is set via thermal poling and retained upon cooling; applied signal voltages enable push–pull modulation. (**c**) Setup for 40 Gbit/s data transmission using an amplified electrical signal and optical carrier, with digital filtering at the receiver. (**d**) Eye diagrams for Device 5 after 330 h and Device 2 after 2700 h high-temperature storage show minimal signal degradation; Device 2 requires ~10% higher drive voltage [191].

Kieninger et al. demonstrated Si-organic hybrid (SOH) modulators that maintain long-term thermal stability, meeting Telcordia reliability standards for high-temperature operation [191]. These devices incorporated an organic EO polymer with a glass transition temperature of 172 °C. Figure 12a,b illustrates the structure of an SOH Mach–Zehnder modulator (MZM). In Figure 12a, the top view shows two multi-mode interference (MMI) couplers used to split and recombine the optical signal. The modulator integrates a ground-signal-ground (GSG) coplanar transmission line for driving the radio frequency (RF) signal across the device. Figure 12b presents a perspective view of the MZM arms, each composed of an Si slot waveguide formed by two closely spaced rails filled with an organic electro-optic (OEO) material. Thin n-doped Si slabs and electrical vias connect the slot waveguides to the aluminum GSG electrodes. The RF drive voltage was concentrated across the narrow slot, generating strong electric fields in the OEO region. This, combined with the confinement of the optical mode in the same slot, results in a high modulation efficiency. EO activity was established through a one-time poling process, where the device was heated near the glass transition temperature of the OEO material, and a voltage is applied to align dipolar molecules. Once cooled, this alignment is fixed, allowing a push–pull operation via differential phase shifts in the two arms of the modulator.

The experimental setup is shown in Figure 12c. On–off keying (OOK) eye diagrams at 40 Gbit/s for Device 5 (330 h) and Device 2 (2700 h) show a π-voltage increase from 2.0 V to 2.2 V (Figure 12d). To maintain similar signal quality, Device 5 was driven at 1.7 Vpp (Q-factor = 8.9), while Device 2 required 1.9 Vpp (Q-factor = 8.3). The 10% increase in drive voltage corresponds to the higher π-voltage of Device 2, indicating that high-temperature storage primarily affects the π-voltage without altering high-speed performance. The modulators continue to deliver high-performance operation, as confirmed by the generation of 40 Gbit/s on–off keying signals after both burn-in and long-term thermal storage [191].

## 7. Emerging Trends and Future Directions

The field of TO and EO materials is experiencing a paradigm shift, driven by the increasing demand for highly integrated, energy-efficient, and dynamically reconfigurable photonic systems. A prominent trajectory in current research is the integration of functional, tunable materials with Si photonics and PICs [192]. While Si offers a well-established, CMOS-compatible platform with high fabrication maturity, the absence of a strong EO effect and the limitations of TO tuning constrain its use in high-speed, actively tunable applications [193]. To address this limitation, significant efforts have been directed toward the heterogeneous or hybrid integration of materials exhibiting strong Pockels or TO effects, such as LN [70,107,194], BTO [195,196], and EO polymers [197,198] onto Si substrates. This approach enables the realization of high-speed modulators, low-power switches, and thermally reconfigurable waveguide components. Recent progress in wafer bonding [199,200], thin-film deposition [201], and patterning techniques [202,203,204] has facilitated the development of monolithically integrated photonic circuits with enhanced tunability, bandwidth, and operational stability [106].

EO and TO materials-based photonic devices have seen significant advancements in recent years, driven by the growing demand for high-speed, low-power optical communication and integrated photonic systems. EO devices are benefiting from the development of materials like lithium niobate on insulator (LNOI) and polymers with high EO coefficients, enabling compact, efficient modulators and switches [205]. TO devices continue to be widely used in tunable filters, phase shifters, and optical switches, particularly in Si photonics platforms, due to their simplicity and compatibility with CMOS fabrication [206,207]. However, TO devices typically suffer from slower response times and higher power consumption compared to their EO counterparts. Ongoing research focuses on improving the material properties, integration techniques, and thermal management strategies to enhance performance, paving the way for more efficient, scalable, and versatile PICs.

Hyperbolic materials and nanoparticles are emerging as promising components for TO tunable photonic devices due to their unique ability to manipulate light at subwavelength scales [208,209]. Hyperbolic materials, characterized by extreme anisotropy in their permittivity tensor, support high-density optical states and enable strong light–matter interactions, which can be highly sensitive to temperature variations. When integrated into photonic structures, hyperbolic metamaterials and nanoparticles, such as those made from doped semiconductors or metal-dielectric composites, can exhibit enhanced TO responses, allowing the efficient and compact thermal tuning of optical properties [210]. Their nanoscale dimensions also support rapid thermal diffusion, potentially improving the modulation speed compared to bulk materials [211].

Concurrently, the application of artificial intelligence (AI) and machine learning (ML) methodologies is revolutionizing the discovery and optimization of tunable photonic materials [212,213]. Data-driven approaches and high-throughput screening, combined with inverse design algorithms, enable the prediction and tailoring of key material parameters such as refractive index modulation, EOCs, dielectric breakdown strength, and thermal conductivity across vast compositional spaces [214]. These techniques not only accelerate the identification of novel materials with targeted performance metrics but also support the optimization of synthesis and processing conditions. Furthermore, AI-assisted process control during fabrication emerges as a powerful tool for minimizing variability, enhancing yields, and enabling adaptive tuning in complex photonic architectures [215,216].

Another critical direction involves the development of materials that exhibit high environmental stability and low energy consumption. Many conventional tunable materials, including liquid crystals and phase-change compounds, suffer from limitations such as thermal instability, volatility, and elevated switching thresholds [217,218]. Consequently, recent research has focused on engineering new classes of hybrid organic–inorganic materials, doped polymers, and chemically stabilized perovskites that exhibit robust performance under ambient environmental conditions. These materials are designed to retain tunability while minimizing power consumption and ensuring long-term operational reliability [219]. This is particularly crucial for emerging applications in portable and wearable photonics, remote sensing, and distributed optical systems, where energy efficiency and environmental resilience are essential [220].

In parallel, the advent of additive manufacturing techniques, including multi-material 3D printing, has opened new pathways for fabricating complex, lightweight, and flexible photonic structures incorporating tunable materials [221,222]. The ability to deposit functional materials with precise spatial control enables the construction of conformal and non-planar photonic elements, which are essential for applications in soft robotics, biomedical devices, and embedded optical systems. Moreover, the incorporation of EO and TO materials into printable inks has facilitated the development of reconfigurable photonic circuits and devices with spatially localized tuning capabilities. These developments represent a significant step toward the customizable, low-cost, and scalable manufacturing of integrated photonic systems.

The application landscape for tunable photonic materials continues to expand, encompassing domains such as light detection and ranging (LiDAR), neuromorphic photonics, and quantum information processing [223,224]. In LiDAR, solid-state beam steering using tunable photonic components enables high-speed, vibration-resistant systems with an improved resolution and field-of-view. In neuromorphic computing, EO materials with nonlinear and memory-like behavior are being employed to emulate synaptic functionalities in photonic neural networks, offering pathways toward ultra-fast, low-power computation [225]. In the context of quantum photonics, materials capable of precise and rapid refractive index modulation are critical for the realization of tunable quantum gates, on-demand photon sources, and dynamically reconfigurable quantum circuits [226]. As these emerging applications impose increasingly stringent requirements on material performance, the continued development of advanced tunable materials will play a pivotal role in the future of photonic technologies.

## 8. Conclusions

TO and EO materials have emerged as essential building blocks for tunable photonic devices, enabling the dynamic modulation and control of optical signals across a broad spectrum of applications. These materials underpin a wide range of technologies, including optical switches, modulators, filters, sensors, and reconfigurable waveguides, which are foundational to telecommunications, data centers, biomedical imaging, and emerging quantum photonic systems. TO materials offer the advantages of simplicity, compatibility with various platforms, including Si and polymer photonics, and ease of fabrication. However, their reliance on thermal diffusion inherently limits their speed and energy efficiency. Despite these challenges, they remain indispensable in scenarios where modulation bandwidth is secondary to fabrication scalability and cost-effectiveness.

EO materials, particularly those exhibiting the Pockels effect, provide ultrafast response times and high-precision refractive index control, making them ideal for high-frequency signal processing and advanced modulation formats. Materials such as LN, BTO, and certain organic chromophores are at the forefront of these developments. Nevertheless, challenges persist in achieving low-voltage operation, stable long-term performance, and compatibility with complementary metal oxide semiconductor fabrication processes. A key trend in the field is the growing interest in hybrid integration, where multiple materials and tuning mechanisms are combined to optimize performance across multiple metrics. For instance, incorporating PCMs, two-dimensional semiconductors, or nanostructured metamaterials can offer nonvolatile tuning, enhanced confinement, and novel nonlinear responses. Furthermore, the integration of AI and ML algorithms with photonic circuits may enable smarter, adaptive tuning and system-level optimization.

Future progress will depend on addressing several critical challenges: developing low-loss and CMOS-compatible materials, improving the stability and repeatability of tuning responses, and ensuring scalable fabrication for commercial deployment. Research into novel material systems, such as lead-free perovskites, graphene-based composites, and topological photonic materials, holds significant promise for overcoming the existing tradeoffs among speed, power consumption, and integration complexity.

To conclude, the convergence of TO and EO technologies, supported by interdisciplinary advances in material science, nanofabrication, and device engineering, is paving the way for the next generation of agile and programmable photonic systems. These technologies will be central to the realization of intelligent optical networks, compact lab-on-chip systems, and scalable quantum photonic architectures. Continued innovation and collaboration across academic, industrial, and governmental research sectors will be vital to unlock the full potential of tunable photonic devices in the years ahead.

## Data Availability

No new data were created or analyzed in this study. Data sharing is not applicable to this article.

## Abbreviations

Thermo-optic = TO; Electro-optic = EO, Lithium niobate = LN; Silicon = Si; Silicon nitride = SiN; Thermo-optic coefficient = TOC; Electro-optic coefficient = EOC; Phase change material = PCM; Barium titanate = BTO; Complementary metal-oxide semiconductor = CMOS; Mach–Zehnder interferometer = MZI; Microring resonator = MRR; Photonic integrated circuit = PIC; Metasurfaces = MSs.
